# Viewpoints on Medical Image Processing: From Science to Application

**DOI:** 10.2174/1573405611309020002

**Published:** 2013-05

**Authors:** Thomas M. Deserno (né Lehmann), Heinz Handels, Klaus H. Maier-Hein (né Fritzsche), Sven Mersmann, Christoph Palm, Thomas Tolxdorff, Gudrun Wagenknecht, Thomas Wittenberg

**Affiliations:** 1 Department of Medical Informatics, Uniklinik RWTH Aachen, Germany;; 2 Institute of Medical Informatics, University of Lübeck, Germany;; 3 Medical and Biological Informatics, German Cancer Research Center, Heidelberg, Germany;; 4 Medical and Biological Informatics, Junior Group Computer-assisted Interventions, German Cancer Research Center, Heidelberg, Germany;; 5 Regensburg – Medical Image Computing (Re-MIC), Faculty of Computer Science and Mathematics, Regensburg University of Applied Sciences, Regensburg, Germany;; 6 Institute of Medical Informatics, Charité - Universitätsmedizin Berlin, Germany;; 7 Electronic Systems (ZEA-2), Central Institute of Engineering, Electronics and Analytics, Forschungszentrum Jülich GmbH, Germany;; 8 Image Processing & Biomedical Engineering Department, Fraunhofer Institute for Integrated Circuits IIS, Erlangen, Germany

**Keywords:** Medical imaging, Image processing, Image analysis, Visualization, Multi-modal imaging, Diffusion-weighted imaging, Model-based imaging, Registration, Digital endoscopy, Virtual reality, Robotics.

## Abstract

Medical image processing provides core innovation for medical imaging. This paper is focused on recent developments from science to applications analyzing the past fifteen years of history of the proceedings of the German annual meeting on medical image processing (BVM). Furthermore, some members of the program committee present their personal points of views: (i) multi-modality for imaging and diagnosis, (ii) analysis of diffusion-weighted imaging, (iii) model-based image analysis, (iv) registration of section images, (v) from images to information in digital endoscopy, and (vi) virtual reality and robotics. Medical imaging and medical image computing is seen as field of rapid development with clear trends to integrated applications in diagnostics, treatment planning and treatment.

## 1. INTRODUCTION

Current advances in medical imaging are made in fields such as instrumentation, diagnostics, and therapeutic applications and most of them are based on imaging technology and image processing. In fact, medical image processing has been established as a core field of innovation in modern health care [1] combining medical informatics, neuro-informatics and bioinformatics [2]. 

In 1984, the Society of Photo-Optical Instrumentation Engineers (SPIE) has launched a multi-track conference on medical imaging, which still is considered as the core event for innovation in the field [Methods]. Analogously in Germany, the workshop “Bildverarbeitung für die Medizin (BVM)” (Image Processing for Medicine) has recently celebrated its 20^th^ annual performance. The meeting has evolved over the years to a multi-track conference on international standard [3, 4, 5, 6, 7, 8, 9]. 

Nonetheless, it is hard to name the most important and innovative trends within this broad field ranging from image acquisition using novel imaging modalities to information extraction in diagnostics and treatment. Ritter *et al.* recently emphasized on the following aspects: (i) enhancement, (ii) segmentation, (iii) registration, (iv) quantification, (v) visualization, and (vi) computer-aided detection (CAD) [10].

Another concept of structuring is here referred to as the “from-to” approach. For instance,

- *From nano to macro*: Co-founded in 2002 by Michael Unser of EPFL, Switzerland, The Institute of Electrical and Electronics Engineers (IEEE) has launched an international symposium on biomedical imaging (ISBI). This conference is focused in the motto from nano to macro covering all aspects of medical imaging from sub-cellular to the organ level.
- *From production to sharing*: Another “from-to” migration is seen in the shift from acquisition to communication [11]. Clark *et al.* expected advances in the medical imaging fields along the following four axes: (i) image production and new modalities; (ii) image processing, visualization, and system simulation; (iii) image management and retrieval; and (iv) image communication and telemedicine.
- *From kilobyte to terabyte*: Deserno *et al.* identified another “from-to” migration, which is seen in the amount of data that is produced by medical imagery [12]. Today, High-resolution CT reconstructs images with 8000 x 8000 pixels per slice with 0.7 μm isotropic detail detectability, and whole body scans with this resolution reach several Gigabytes (GB) of data load. Also, microscopic whole-slide scanning systems can easily provide so-called virtual slices in the rage of 30.000 x 50.000 pixels, which equals 16.8 GB on 10 bit gray scale.
- *From science to application*: Finally, in this paper, we aim at analyzing recent advantages in medical imaging on another level. The focus is to identify core fields fostering transfer of algorithms into clinical use and addressing gaps still remaining to be bridged in future research.

The remainder of this review is organized as follows. In Section 3, we briefly analyze the history of the German workshop BVM. More than 15 years of proceedings are currently available and statistics is applied to identify trends in content of conference papers. Section 4 then provides personal viewpoints to challenging and pioneering fields. The results are discussed in Section 5.

## 2. THE GERMAN HISTORY FROM SCIENCE TO APPLICATION

Since 1994, annual proceedings of the presented contributions from the BVM workshops have been published, which are available electronically in postscript (PS) or the portable document format (PDF) from 1996. Disregarding the type of presentation (oral, poster, or software demonstration), the authors are allowed to submit papers with a length of up to five pages. In 2012 the length was increased to six pages. Both, English and German papers are allowed. The number of English contributions increased steadily over the years, and reached about 50% in 2008 [8]. 

**Fig. (1):**
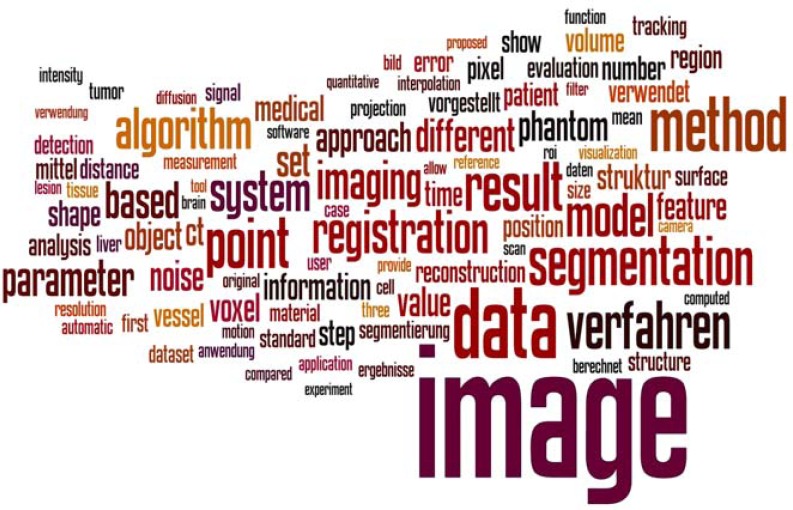
Word cloud representing the most frequent 100 terms counted from the 469 page long BVM proceedings 2012 [13].

In order to analyze the content of the on average 124k words long proceedings regarding the most relevant topics that were discussed on the BVM workshops, the incidence of the most frequent words has been assessed for each proceeding from 1996 until 2012. From this investigation, about 300 common words of the German and English language (e.g. and / und, etc.) have been excluded. (Fig. **1**) presents a word cloud computed from the 100 most frequent terms used in the proceedings of the 2012 BVM workshop. The font sizes of the words refer to their counted frequency in the text. 

It can be seen, in 2012, “image” was the most frequent word occurring in the BVM proceedings (920 incidences), as also observed in all the other years (1996-2012: 10,123 incidences). Together with terms like “reconstruction”, “analysis”, or “processing”, medical imaging is clearly recognizable as the major subject of the BVM workshops.

**Fig. (2):**
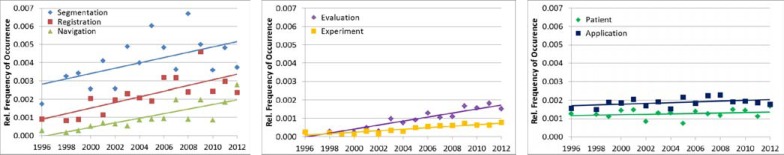
Trends from BVM workshop proceedings from important terms of processing procedures (left), experimental verification (middle), and application to humans (right).

Concerning the scientific direction of the BVM meeting over time, terms such as “segmentation”, “registration”, and “navigation”, which indicate image processing procedures relevant for clinical applications, have been used with increasing frequencies (Fig. **2**, left). The same holds for terms like “evaluation” or “experiment”, which are related to the validation of the contributions (Fig. **2**, middle), constituting a first step towards the transition of the scientific results into a clinical application. (Fig. **2** right) shows the occurrence of the words “patient” and “application” in the contributed papers of the BVM workshops between 1996 and 2012. Here, rather constant numbers of occurrences are found indicating a stringent focus on clinical applications. 

## 3. VIEWPOINTS FROM SCIENCE TO APPLICATION

### 3.1. Multi-modal Image Processing for Imaging and Diagnosis

**Fig. (3):**
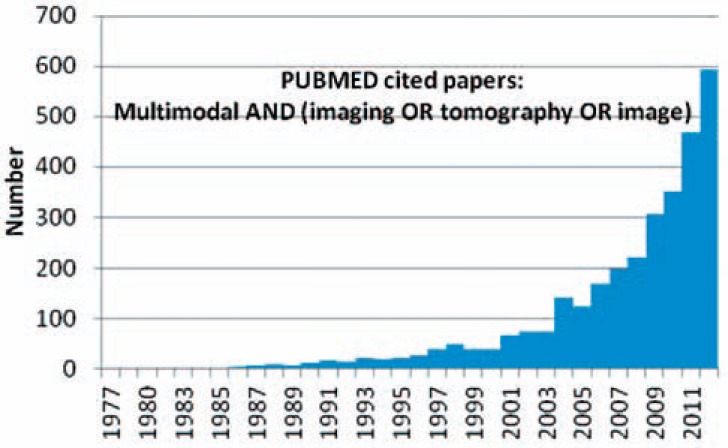
PubMed cited papers for search “multimodal AND (imaging OR tomography OR image)”.

**Fig. (4):**
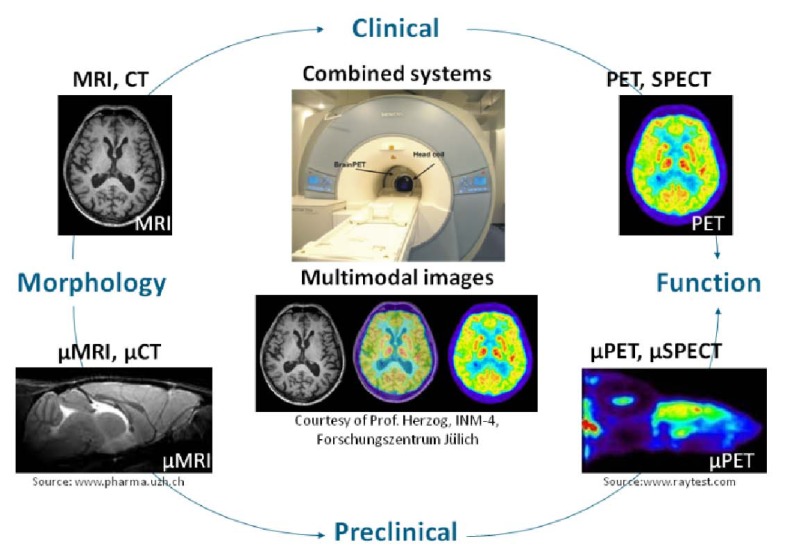
Morphological and functional imaging in clinical and pre-clinical applications.

Multi-modal imaging refers to (i) different measurements at a single tomographic system (e.g., MRI and functional MRI), (ii) measurements at different tomographic systems (e.g., computed tomography (CT), positron emission tomography (PET), and single photon emission computed tomography (SPECT)), and (iii) measurements at integrated tomographic systems (PET/CT, PET/MR). Hence, multi-modal tomography has become increasingly popular in clinical and preclinical applications (Fig. **3**) providing images of morphology and function (Fig. **4**). 

Multi-modal image processing for enhancing multi-modal imaging procedures primarily deals with image reconstruction and artifact reduction. Examples are the integration of additional information about tissue types from MRI as an anatomical prior to the iterative reconstruction of PET images [14] and the CT- or MR-based correction of attenuation artifacts in PET, respectively, which is an essential prerequisite for quantitative PET analysis [15, 16]. Since these algorithms are part of the imaging workflow, only highly automated, fast, and robust algorithms providing adequate accuracy are appropriate solutions. Accordingly, the whole image in the different modalities must be considered.

This requirement differs for multi-modal diagnostic approaches. In most applications, a single organ or parts of an organ are of interest. Anatomical and particularly pathological regions often show a high variability due to structure, deformation, or movement, which is difficult to predict and is thus a great challenge for image processing. In multi-modality applications, images represent complementary information often obtained at different time-scales introducing additional complexity for algorithms. Other inequalities are introduced by the different resolutions and fields of view showing the organ of interest in different degrees of completeness. From a scientific and thus algorithmic point of view, image processing methods for multi-modal images must meet higher requirements than those applied to single-modality images. 

Looking exemplarily at segmentation as one of the most complex and demanding problems in medical image processing, the modality showing anatomical and pathological structures in high resolution and contrast (e.g., MRI, CT) is typically used to segment the structure or volume of interest (VOI) to subsequently analyze other properties such as function within these target structures. Here, the different resolutions have to be regarded to correct for partial volume effects in the functional modality (e.g., PET, SPECT). Since the structures to be analyzed are dependent on the disease of the actual patient examined, automatic segmentation approaches are appropriate solutions if the anatomical structures of interest are known beforehand [17], while semi-automatic approaches are advantageous if flexibility is needed [18, 19].

Transferring research into diagnostic application software requires a graphical user interface (GUI) to parameterize the algorithms, 2D and 3D visualization of multi-modal images and segmentation results, and tools to interact with the visualized images during the segmentation procedure. The Medical Interaction Toolkit [20] or the MevisLab [21] provide the developer with frameworks for multi-modal visualization, interaction and tools to build appropriate GUIs, yielding an interface to integrate new algorithms from science to application. 

Another important aspect transferring algorithms from pure academics to clinical practice is evaluation. Phantoms can be used for evaluating specific properties of an algorithm, but not for evaluating the real situation with all its uncertainties and variability. Thus, the most important step of migrating is extensive testing of algorithms on large amounts of real clinical data, which is a great challenge particularly for multi-modal approaches, and should in future be more supported by publicly available databases.

### 3.2. Analysis of Diffusion Weighted Images

Due to its sensitivity to micro-structural changes in white matter, diffusion weighted imaging (DWI) is of particular interest to brain research. Stroke is the most common and well known clinical application of DWI, where the images allow the non-invasive detection of ischemia within minutes of onset and are sensitive and relatively specific in detecting changes triggered by strokes [22]. The technique has also allowed deeper insights into the pathogenesis of Alzheimer’s disease, Parkinson disease, autism spectrum disorder, schizophrenia, and many other psychiatric and non-psychiatric brain diseases. DWI is also applied in the imaging of (mild) traumatic brain injury, where conventional techniques lack sensitivity to detect the subtle changes occurring in the brain. Here, studies on sports-related traumata in the younger population have raised considerable debates in the recent past [23]. 

Methodologically, recent advances in the generation and analysis of large-scale networks on basis of DWI are particularly exciting and promise new dimensions in quantitative neuro-imaging via the application of the profound set of tools available in graph theory to brain image analysis [24]. DWI sheds light on the living brain network architecture, revealing the organization of fiber connections together with their development and change in disease. 

Big challenges remain to be solved though: Despite many years of methodological development in DWI post-processing, the field still seems to be in its infancy. The reliable tractography-based reconstruction of known or pathological anatomy is still not solved. Current reconstruction challenges at the 2011 and 2012 annual meetings of the Medical Image Computing and Computer Assisted Intervention (MICCAI) Society have demonstrated the lack of methods that can reliably reconstruct large and well-known structures like the cortico-spinal tract in datasets of clinical quality [25]. Missing reference-based evaluation techniques hinder the well-founded demonstration of the real advantages of novel tractography algorithms over previous methods [26]. The mentioned limitations have obscured a broader application of DWI tractography, e.g. in surgical guidance. Even though the application of DWI e.g. in surgical resection has shown to facilitate the identification of risk structures [27], the widespread use of these techniques in surgical practice remains limited mainly by the lack of robust and standardized methods that can be applied multi-centered across institutions and comprehensive evaluation of these algorithms.

**Fig. (5):**
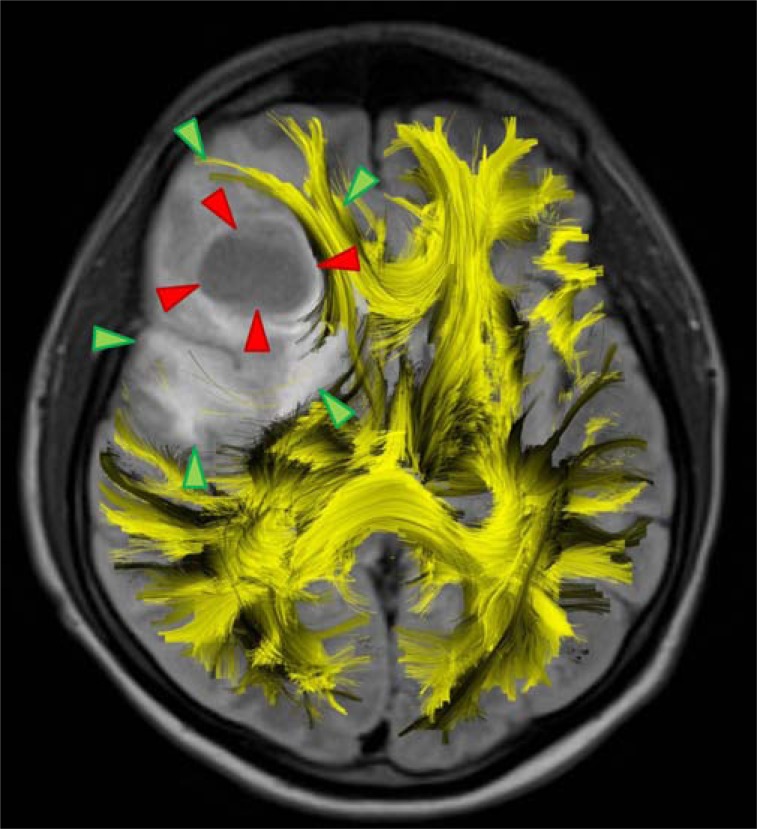
Depiction of fiber tracts in the vicinity of a grade IV glioblastoma. The volumetric tracking result (yellow) was overlaid on an axial T2-FLAIR image. Red and green arrows indicate the necrotic tumor core and peritumoral hyperintensity, respectively. In the frontal parts, fiber tracts are still depicted, whereas in the dorsal part, tracts seem to be either displaced or destructed by the tumor.

However, there are numerous applications of DWI in cancer imaging, which bridge imaging science and clinical application. The imaging modality has shown potential in the detection, staging and characterization of tumors (Fig. **5**), the evaluation of therapy response, or even in the prediction of therapy outcome [28]. DWI was also applied in the detection and characterization of lesions in the abdomen and the pelvis, where increased cellularity of malignant tissue leads to restricted diffusion when compared to the surrounding tissue [29]. The challenge here again will be the establishment of reliable sequences and post-processing methods for the wide-spread and multi-centric application of the techniques in the future.

### 3.3. Model-Based Image Analysis

As already emphasized in the previous viewpoints, there is a big gap between the state of the art in current research and methods available in clinical application, especially in the field of medical image analysis [30]. Segmentation of relevant image structures (tissues, tumors, vessels etc.) is still one of the key problems in medical image computing lacking robust and automatic methods. The application of pure data-driven approaches like thresholding, region growing, edge detection, or enhanced data-driven methods like watershed algorithms, Markov random field (MRF)-based approaches, or graph cuts often leads to weak segmentations due to low contrasts between neighboring image objects, image artifacts, noise, partial volume effects etc. 

Model-based segmentation integrates a-priori knowledge of the shapes and appearance of relevant structures into the segmentation process. For example, the local shape of a vessel can be characterized by the vesselness operator [31], which generates images with an enhanced representation of vessels. Using the vesselness information in combination with the original grey value image segmentation of vessels can be improved significantly and especially the segmentation of a small vessel becomes possible (e.g. [32]). 

In statistical or active shape and appearance models [33, 34], shape variability in organ distribution among individuals and characteristic gray value distributions in the neighborhood of the organ can be represented. In these approaches, a set of segmented image data is used to train active shape and active appearance models, which include information about the mean shape and shape variations as well as characteristic gray value distributions and their variation in the population represented in the training data set. Instead of direct point-to-point correspondences that are used during the generation of classical statistical shape models, Hufnagel *et al.* have suggested probabilistic point-to-point correspondences [35]. This approach takes into account that often inaccuracies are unavoidable by the definition of direct point correspondences between organs of different persons. In probabilistic statistical shape models, these correspondence uncertainties are respected explicitly to improve the robustness and accuracy of shape modeling and model-based segmentation. Integrated in an energy minimizing level set framework, the probabilistic statistical shape models can be used for enhanced organ segmentation [36]. 

In contrast thereto, atlas-based segmentation methods (e.g., [37]) realize a case-based approach and make use of the segmentation information contained in a single segmented data set, which is transferred to an unseen patient image data set. The transfer of the atlas segmentation to the patient segmentation is done by inter-individual non-linear registration methods. Multi-atlas segmentation methods using several atlases have been proposed (e.g. [38]) and show an improved accuracy and robustness in comparison to single atlas segmentation methods. Hence, multi-atlas approaches are currently in the focus of further research [39, 40].

In future, more task-oriented systems integrated into diagnostic processes, intervention planning, therapy and follow-up are needed. In the field of image analysis, due the limited time of the physicians, automatic procedures are of special interest to segment and extract quantitative object parameters in an accurate, reproducible and robust way. Furthermore, intelligent and easy-to-use methods for fast correction of unavoidable segmentation errors are needed.

### 3.4. Registration of Section Images

Imaging techniques such as histology [41] or auto-radiography [42] are based on thin post-mortem sections. In comparison to *in-vivo* imaging, e.g. positron emission tomography (PET), magnetic resonance imaging (MRI), or DWI (as addressed in the previous viewpoint, cf. Section 4.1), several properties are considered advantageous. For instance, tissue can be processed after sectioning to enhance contrast (e.g. staining) [43], to mark specific properties like receptors [44] or to apply laser ablation studying the spatial element distribution [45]; tissue can be scanned in high-resolution [43]; and tissue is thin enough to allow optical light transmission imaging, e.g. polarized light imaging (PLI) [46]. Therefore, section imaging results in high space-resolved and high-contrasted data, which supports findings such as cytoarchitectonic boundaries [47], neuronal fiber directions [48], and receptor or element distributions [45]. 

Restacking of 2D sections into a 3D volume followed by the fusion of this stack with an in-vivo volume is the challenging task of medical image processing on the track from science to application. The 3D section stacks then serve as an atlas for a large variety of applications. Sections are non-linearly deformed during cutting and post-processing. Additionally, discontinuous artifacts like tears or enrolled tissue hamper the correspondence of true structure and tissue imaged. 

The so-called “problem of the digitized banana” [41] prohibits the section-by-section registration without 3D reference. Smoothness of registered stacks is not equivalent to consistency and correctness. Whereas the deformations are section-specific, the orientation of the sections in comparison to the 3D structure depends on the cutting direction and, thus, is the same for all sections. In this tangled situation the question rises, if it is better to (i) restack the sections first, register the whole stack afterwards and correct for deformations at last (volume-first approach) or (ii) to register each section individually to the 3D reference volume while correcting deformations at the same time (section-first approach). Both approaches combine

**Fig. (6):**
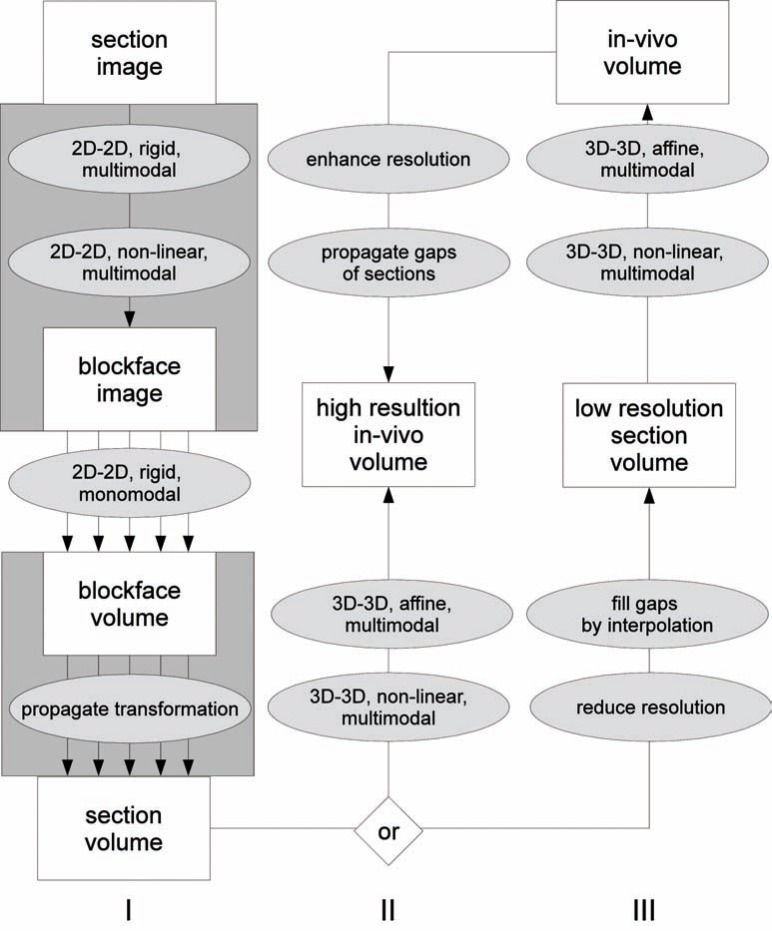
Characteristic flow chart of volume-first approach and volume generation with (gray boxes) or without blockface images as intermediate reference modality (Column I). Either the in-vivo volume is post-processed to generate a pseudo-high-resolution volume with propagated section gaps (Column II) or the section volume is post-processed to get a low-resolution stack with filled gaps (Column III) [42].

- *Multi-modal registration*: The need of a 3D reference and the application to correlate high-resolution section imaging findings with in-vivo imaging are sometimes solved at the same time. If possible, the 3D in-vivo modality itself is used as a reference.
- *Multi-resolution registration*: One of the most interesting features of section imaging is its high resolution in the *x*- and *y*-direction. The *z*-resolution is determined by the section thickness (25 μm – 150 μm) and the number of sections. Registration has to address non-equidistant sectioning (Fig. **6**).

Due to the variety of difficulties, missing evaluation possibilities and section specifics like post-processing, embedding, cutting procedure and tissue type there is not just one best approach to come from 2D to 3D. But careful work in this field is paid off by cutting edge applications. Not least within the European flagship, The Human Brain Project (HBP), further research in this area of medical image processing is demanded. The state-of-the-art review of HBP states in the context of human brain mapping: “What is missing to date is an integrated open source tool providing a standard application programming interface (API) for data registration and coordinate transformations and guaranteeing multi-scale and multi-modal data accuracy” [49]. Such a tool will narrow the gap from science to application.

### 3.5. From Images to Information in Digital Endoscopy 

**Fig. (7):**
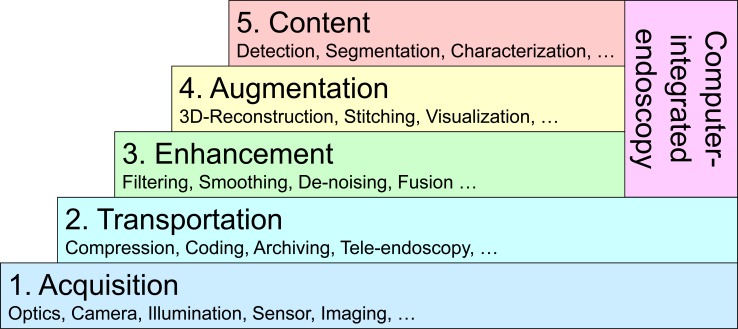
Modules to build computer-integrated endoscopy, which enables information gain from image data.

Basic endoscopic technologies and their routine applications (Fig. **7**, bottom layers) still are purely data-oriented, as the complete image analysis and interpretation is performed solely by the physician. If content of endoscopic imagery is analyzed automatically, several new application scenarios for diagnostics and intervention with increasing complexity can be identified (Fig. **7**, upper layers). As these new possibilities of endoscopy are inherently coupled with the use of computers, these new endoscopic methods and applications can be referred to as computer-integrated endoscopy [50]. Information, however, is referred to on the highest of the five levels of semantics (Fig. **7**):

- 1.* Acquisition*: Advancements in diagnostic endoscopy were obtained by glass fibers for the transmission of electric light into and image information out of the body. Besides the pure wire-bound transmission of endoscopic imagery, in the past 10 years wireless broadcast came available for gastroscopic video data captured from capsule endoscopes [51].
- 2.* Transportation*: Based on digital technologies, essential basic processes of endoscopic still image and image sequence capturing, storage, archiving, documentation, annotation and transmission have been simplified. These developments have initially led to the possibilities for tele-diagnosis and tele-consultations in diagnostic endoscopy, where the image data is shared using local networks or the internet [52].
- 3.* Enhancement*: Methods and applications for image enhancement include intelligent removal of honey-comb patterns in fiberscopic recordings [53], temporal filtering for the reduction of ablation smoke and moving particles [54], image rectification for gastroscopes. Additionally, besides having an increased complexity, they have to work in real time with a maximum delay of 60 milliseconds, to be acceptable for surgeons and physicians.
- 4.* Augmentation*: Image processing enhances endoscopic views with additional type of information. Examples of this type are artificial working horizon, key-hole views to endoscopic panorama-images [55], 3D surfaces computed from point clouds obtained by special endoscopic imaging devices such as stereo endoscopes [56], time-of-flight endoscopes [57], or shape-from polarization approaches [58]. This level also includes the possibilities of visualization and image fusion of endoscopic views with preoperative acquired radiological imagery such as angiography or CT data [59] for better intra-operative orientation and navigation, as well as image-based tracking and navigation through tubular structures [60].
- 5.* Content*: Methods of content-based image analysis consider the automated segmentation, characterization and classification of diagnostic image content. Such methods describe computer-assisted detection (CADe) [61] of lesions (such as e.g. polyps) or computer-assisted diagnostics (CADx) [62], where already detected and delineated regions are characterized and classified into, for instance, benign or malign tissue areas. Furthermore, such methods automatically identify and track surgical instruments, e.g. supporting robotic surgery approaches.

On the technical side the semantics of the extracted image contents increases from the pure image recording up to the image content analysis level. This complexity also relates to the expected time axis needed to bring these methods from science to clinical applications. 

From the clinical side, the most complex methods such as automated polyp detection (CADe) are considered as most important. However, it is expected that computer-integrated endoscopy systems will increasingly enter clinical applications and as such will contribute to the quality of the patient’s healthcare. 

### 3.6. Virtual Reality and Robotics

Virtual reality (VR) and robotics are two rapidly expanding fields with growing application in surgery. VR creates three-dimensional environments increasing the capability for sensory immersion, which provides the sensation of being present in the virtual space. Applications of VR include surgical planning, case rehearsal, and case playback, which could change the paradigm of surgical training, which is especially necessary as the regulations surrounding residencies continue to change [63]. Surgeons are enabled to practice in controlled situations with preset variables to gain experience in a wide variety of surgical scenarios [64].

With the availability of inexpensive computational power and the need for cost-effective solutions in healthcare, medical technology products are being commercialized at an increasingly rapid pace. VR is already incorporated into several emerging products for medical education, radiology, surgical planning and procedures, physical rehabilitation, disability solutions, and mental health [65]. For example, VR is helping surgeons learn invasive techniques before operating, and allowing physicians to conduct real-time remote diagnosis and treatment. Other applications of VR include the modeling of molecular structures in three dimensions as well as aiding in genetic mapping and drug synthesis.

In addition, the contribution of robotics has accelerated the replacement of many open surgical treatments with more efficient minimally invasive surgical techniques using 3D visualization techniques. Robotics provides mechanical assistance with surgical tasks, contributing greater precision and accuracy and allowing automation. Robots contain features that can augment surgical performance, for instance, by steadying a surgeon’s hand or scaling the surgeon’s hand motions [66]. Current robots work in tandem with human operators to combine the advantages of human thinking with the capabilities of robots to provide data, to optimize localization on a moving subject, to operate in difficult positions, or to perform without muscle fatigue. Surgical robots require spatial orientation between the robotic manipulators and the human operator, which can be provided by VR environments that re-create the surgical space. This enables surgeons to perform with the advantage of mechanical assistance but without being alienated from the sights, sounds, and touch of surgery [67]. 

After many years of research and development, Japanese scientists recently presented an autonomous robot which is able to realize surgery within the human body [68]. They send a miniature robot inside the patient’s body, perceive what the robot saw and touched before conducting surgery by using the robot’s minute arms as though as it were the one’s of the surgeon.

While the possibilities – and the need – for medical VR and robotics are immense, approaches and solutions using new applications require diligent, cooperative efforts among technology developers, medical practitioners and medical consumers to establish where future requirements and demand will lie. Augmented and virtual reality substituting or enhancing the reality can be considered as multi-reality approaches [69], which are already available in commercial products for clinical applications.

## 4. DISCUSSION

In this paper, we have analyzed the written proceedings of the German annual meeting on Medical Imaging (BVM) and presented personal viewpoints on medical image processing focusing on the transfer from science to application. Reflecting successful clinical applications and promising technologies that have been recently developed, it turned out that medical image computing has transferred from single- to multi-images, and there are several ways to combine these images:

- *Multi-modality*: Figs. **2** and **3** have emphasized that medical image processing has been moved away from the simple 2D radiograph via 3D imaging modalities to *multi-modal* processing and analyzing. Successful applications that are transferrable into the clinics jointly process imagery from different modalities.
- *Multi-resolution*: Here, images with different properties from the same subject and body area need alignment and comparison. Usually, this implies a multi-resolution approach, since different modalities work on different scales of resolutions.
- *Multi-scale*: If data becomes large, as pointed out for digital pathology, algorithms must operate on different scales, iteratively refining the alignment from coarse-to-fine. Such algorithmic design usually is referred to as multi-scale approach.
- *Multi-subject*: Models have been identified as key issue for implementing applicable image computing. Such models are used for segmentation, content understanding, and intervention planning. They are generated from a reliable set of references, usually based on several subjects.
- *Multi-atlas*: Even more complex, the personal viewpoints have identified multi-atlas approaches that are nowadays addressed in research. For instance in segmentation, accuracy and robustness of algorithms are improved if they are based on multiple rather than a single atlas. Both, accuracy and robustness are essential requirements for transferring algorithms into the clinical use.
- *Multi-semantics*: Based on the example of digital endoscopy, another “multi” term is introduced. Image understanding and interpretation has been defined on several levels of semantics, and successful applications in computer-integrated endoscopy are operating on several of such levels.
- *Multi-reality*: Finally, our last viewpoint has addressed the augmentation of the physician’s view by means of virtual reality. Medical image computing is applied to generate and superimpose such views, which results in a multi-reality world.

Andriole, Barish, and Khorasani also have discussed issues to consider for advanced image processing in the clinical arena [70]. In completion of the collection of “multi” issues, they emphasized that radiology practices are experiencing a tremendous increase in the number of images associated with each imaging study, due to *multi-slice*, *multi-plane* and/or *multi-detector* 3D imaging equipment. Computer-aided detection used as a second reader or as a first-pass screener will help maintaining or perhaps improving readers' performance on such big data in terms of sensitivity and specificity.

Last not least, with all these “multies”, the computational load of algorithms again becomes an issue. Modern computers provide enormous computational power and yield a revisiting and applications of several “old” approaches, which did not find their way into the clinical use yet, just because of the processing times. However, combining many images of large sizes, processing time becomes crucial again. Scholl *et al.* have recently addressed this issue reviewing applications based on parallel processing and usage of graphical processors for image analysis [12]. These are seen as *multi-processing* methods.

In summary, medical image processing is a progressive field of research, and more and more applications are becoming part of the clinical practice. These applications are based on one or more of the “multi” concepts that we have addressed in this review. However, effects from current trends in the Medical Device Directives that increase the efforts needed for clinical trials of new medical imaging procedure, cannot be observed until today. It will hence be an interesting point to follow the trend of the translation of scientific results of future BVM workshops into clinical applications.
